# Accelerating Onset of Puberty Through Modification of Early Life Nutrition Induces Modest but Persistent Changes in Bull Sperm DNA Methylation Profiles Post-puberty

**DOI:** 10.3389/fgene.2020.00945

**Published:** 2020-08-26

**Authors:** Jean-Philippe Perrier, David A. Kenny, Aurélie Chaulot-Talmon, Colin J. Byrne, Eli Sellem, Luc Jouneau, Anne Aubert-Frambourg, Laurent Schibler, Hélène Jammes, Patrick Lonergan, Sean Fair, Hélène Kiefer

**Affiliations:** ^1^Laboratory of Animal Reproduction, Department of Biological Sciences, Biomaterials Research Cluster, Bernal Institute, University of Limerick, Limerick, Ireland; ^2^Animal and Bioscience Research Department, Animal and Grassland Research and Innovation Centre, Teagasc, Grange, Co. Meath, Ireland; ^3^Université Paris-Saclay, UVSQ, INRAE, BREED, Jouy-en-Josas, France; ^4^Ecole Nationale Vétérinaire d’Alfort, BREED, Maisons-Alfort, France; ^5^R&D Department, ALLICE, Paris, France; ^6^School of Agriculture and Food Science, University College Dublin, Dublin, Ireland

**Keywords:** Bovine, puberty, spermatozoa, DNA methylation, nutritional programming

## Abstract

In humans and model species, alterations of sperm DNA methylation patterns have been reported in cases of spermatogenesis defects, male infertility and exposure to toxins or nutritional challenges, suggesting that a memory of environmental or physiological changes is recorded in the sperm methylome. The objective of this study was to ascertain if early life plane of nutrition could have a latent effect on DNA methylation patterns in sperm produced post-puberty. Holstein-Friesian calves were assigned to either a high (H) or moderate (M) plane of nutrition for the first 24 weeks of age, then reassigned to the M diet until puberty, resulting in HM and MM groups. Sperm DNA methylation patterns from contrasted subgroups of bulls in the HM (ejaculates recovered at 15 months of age; *n* = 9) and in the MM (15 and 16 months of age; *n* = 7 and 9, respectively) were obtained using Reduced Representation Bisulfite Sequencing. Both 15 and 16 months were selected in the MM treatment as these bulls reached puberty approximately 1 month after the HM bulls. Hierarchical clustering demonstrated that inter-individual variability unrelated to diet or age dominated DNA methylation profiles. While the comparison between 15 and 16 months of age revealed almost no change, 580 differentially methylated CpGs (DMCs) were identified between the HM and MM groups. Differentially methylated CpGs were mostly hypermethylated in the HM group, and enriched in endogenous retrotransposons, introns, intergenic regions, and shores and shelves of CpG islands. Furthermore, genes involved in spermatogenesis, Sertoli cell function, and the hypothalamic–pituitary–gonadal axis were targeted by differential methylation when HM and MM groups were compared at 15 months of age, reflecting the earlier timing of puberty onset in the HM bulls. In contrast, the genes still differentially methylated in MM bulls at 16 months of age were enriched for ATP-binding molecular function, suggesting that changes to the sperm methylome could persist even after the HM and MM bulls reached a similar level of sexual maturity. Together, results demonstrate that enhanced plane of nutrition in pre-pubertal calves associated with advanced puberty induced modest but persistent changes in sperm DNA methylation profiles after puberty.

## Introduction

With the advent of genomic selection, genetically elite dairy bulls can now be reliably identified as sires for use in artificial insemination shortly after birth; however, semen cannot be supplied until after puberty is reached. Earlier onset of puberty in young, genomically selected, high genetic merit dairy bulls will advance the availability of semen, shorten the generation interval, and accelerate genetic improvement. Recent data from our group (Byrne et al., 2018a, b) demonstrated that offering bull calves a high plane of nutrition in early life hastens the age at puberty onset by approximately 1 month. Indeed, our own research and that of others (Harstine et al., 2015; Dance et al., 2016) on Holstein-Friesian bulls has clearly shown that an enhanced plane of nutrition up to 6 months of age does not result in any latent effects on measures of post-pubertal semen quality, including pre- and post-thaw sperm motility, or on the *in vitro* fertilizing ability (Dance et al., 2015, 2016), despite leading to increased scrotal skin thickness (Byrne et al., 2018a) compared to their contemporaries offered a moderate plane of nutrition. In addition to these alterations, thermographic imaging of the testes also highlighted differences in the scrotal surface temperature (Byrne et al., 2018a). There is some evidence for a negative effect of increased dietary energy on semen quality in post-pubertal beef bulls, possibly mediated through increased testicular temperatures (Coulter et al., 1997). However, there is a dearth of information on whether such intensive nutritional regimes negatively affect a bull’s post-pubertal semen production when employed in early life. In particular, insight into the biochemical or molecular changes induced by nutrition and affecting sperm cells – in particular those of a more persistent nature which could be transmitted to the next generation – is essential.

Epigenetics refers to the molecular mechanisms that alter gene regulation in a DNA sequence-independent fashion. More specifically, cell differentiation requires a specific transcriptional program that relies on combinations of histone modifications, DNA methylation states and non-coding RNAs. These epigenetic modifications constitute the memory of tissue-specific regulations that are transmitted to the daughter cells through cell divisions (Bird, 2007), and have the potential to record past environmental or physiological changes. The sperm epigenome could therefore provide useful markers of the latent effect of early life nutrition. The production of mature sperm requires sequential and complex reorganization of the epigenomic architecture of sperm cell precursors, which begins during fetal life and is only completed after sexual maturity is reached. Epigenetic reprogramming during fetal development first consists of a genome-wide erasure of DNA methylation marks of primordial germ cells (Popp et al., 2010; von Meyenn and Reik, 2015; Tang et al., 2016). Re-establishment of male germline-specific DNA methylation marks then progressively occurs before birth and during early post-natal life. After puberty and during spermatogenesis, DNA methylation patterns are essentially maintained, whereas profound rearrangements of DNA packaging (histone to protamine replacement, addition of sperm-specific histone variants, and histone post-translational modifications), and significant changes of sperm non-coding RNA content can be observed (Carrell, 2012; McSwiggin and O’Doherty, 2018). Any alterations of these dynamic epigenetic processes by intrinsic or environmental factors can potentially affect fertility, as well as influence the phenotype of the offspring (Chavatte-Palmer et al., 2016; Champroux et al., 2018; Kiefer and Perrier, 2020).

There is growing evidence that physiological, metabolic, and environmental factors, and in particular diet, can have an impact on the sperm methylome. For example, differential DNA methylation patterns in human sperm have been detected between men before and after gastric-bypass induced weight loss (Donkin et al., 2016). Similarly, in the rat, high-fat diet induces modifications in the sperm methylome (de Castro Barbosa et al., 2015), while no diet-related modifications have been found in the sperm of mice challenged with three different diets (control, low protein, and high fat diets; Shea et al., 2015). Depending on the study, mice subjected to low-protein diet or caloric restriction before conception showed either no detectable (Carone et al., 2010) or significant (Martínez et al., 2014; Radford et al., 2014) changes to DNA methylation pattern in their sperm, while a low protein diet during adulthood impacted testicular morphology and lowered the global DNA methylation content of sperm cells (Watkins et al., 2018). In addition, folic acid deficiency resulted in delayed meiotic onset, alterations in the sperm methylome and negative pregnancy outcomes in female mice (Lambrot et al., 2013), while its supplementation induced a decreased sperm count coupled with alterations of DNA methylation pattern of several imprinted genes (Ly et al., 2017). In addition to the direct effects on sperm, recent published studies have demonstrated that alterations of the sperm methylome can potentially affect the offspring metabolic phenotype, such as glucose tolerance or insulin sensitivity (reviewed in Schagdarsurengin and Steger, 2016; Donkin and Barrès, 2018).

We have previously shown that the bovine sperm methylome is unique, being globally hypomethylated compared to that of other mammals (Perrier et al., 2018). While within the context of the livestock industry, a number of studies on DNA methylation patterns have focused on male fertility (Kropp et al., 2017) or on the effects of various types of dietary supplementation regimen (reviewed by Murdoch et al., 2016), none have examined the existence of residual effects of pre-pubertal diet on sperm DNA methylation profiles after puberty in the bull. The aim of this study was to examine the effect of plane of nutrition during the first 6 months of life and advanced puberty on post-pubertal sperm DNA methylation patterns. We also compared bulls between 15 and 16 months of age, as previous work from our group (Byrne et al., 2018a, b) demonstrated that puberty was advanced by approximately 1 month in bulls fed a high plane of nutrition during the first 6 months of life, allowing us to evaluate how the DNA methylation pattern evolves during the weeks following this critical event.

## Materials and Methods

### Animals and Semen Collection

The management of the bulls has been described in detail (Byrne et al., 2018a). Diets were designed using National Research Council (NRC) guidelines (2001). Briefly, autumn-born Holstein-Friesian bull calves were assigned to a high (H) or moderate (M) plane of nutrition for the first 6 months of life. They were offered pre-weaning diets for a minimum of 56 days (corresponding to 247 and 117% of NRC energy and protein requirements for 55 kg calves, for calves on the H and M diets, respectively) and were weaned once they were consuming 1 kg of concentrates for three consecutive days, which happened at a mean (±SD) age of 17 (±4.4) days. After weaning, calves on the H diet received 1200 g of milk replacer and concentrate *ad libitum*, whereas calves on the M diet received 450 g of milk replacer and a maximum of 1 kg of concentrate daily (corresponding to 166 and 98% of NRC energy and protein requirements for 90 kg calves, respectively). All calves were offered grass hay to appetite and had *ad libitum* access to water. At 24 weeks of age, all calves were fed a moderate plane of nutrition consisting of 0.5 kg of concentrate daily and grass to appetite (corresponding to 178% of NRC energy and protein requirements), resulting in the HM and MM groups After puberty, all bulls received 4 kg of concentrates and grass silage *ad libitum* until 60 weeks of age. From 60 weeks of age, all animals were fed concentrate *ad libitum* plus 5 kg of grass silage, until slaughter at 72 weeks of age. The complete composition of diets can be found in Supplementary Table 1 (chemical composition of milk replacer), Supplementary Table 2 (composition and chemical analyses of the concentrate), and Supplementary Table 3 (chemical analysis of forages). To monitor the onset of puberty, animals were weighed weekly pre-weaning and fortnightly post-weaning. The average daily gain pre- and post- weaning can be found in Supplementary Table 4

Semen was collected from the bulls on a bi-weekly basis using electro-ejaculation once their scrotal circumference reached 24 cm. Puberty was defined as an ejaculate with >50 million sperm with a progressive motility of >10% (Wolf et al., 1965) in two consecutive ejaculates. Among the HM and MM bulls, those with the most extreme differences in terms of age at puberty were selected for the present study (*n* = 9 bulls per group). Semen was collected at 15 and 16 months of age via electro-ejaculation, resulting in four groups of samples (Supplementary Table 5): HM15 (*n* = 9), MM15 (*n* = 7), HM16 (*n* = 7), and MM16 (*n* = 9). Semen was diluted in Bioxcell (IMV), to a concentration of 80 × 10^6^ per ml. Straws (0.25 ml) were then frozen to -140°C over a 9 min. period (-15.5°C/min) in a programmable freezer (Planar), followed by immersion and storage in liquid nitrogen, pending further analysis.

### Genomic DNA Extraction

One straw of bull semen was used for DNA extraction (∼20 million sperm). After thawing at 37°C, the semen was washed with phosphate-buffered saline (PBS) to remove the extender, then briefly washed with H_2_O to eliminate somatic cells that may have contaminated the sample, and incubated overnight at 55°C in 200 μl lysis buffer (10 mM Tris-HCl pH 7.5, 25 mM EDTA, 1% SDS, 75 mM NaCl, 50 mM dithiothreitol and 0.5 μg glycogen) in the presence of 0.2 mg/ml proteinase K. Microscopic examination of all samples before lysis revealed no contamination with somatic cells. After incubation with 25 μg/ml RNAse A for 1 h at 37°C, genomic DNA was extracted twice using phenol and phenol:chloroform (1:1), then ethanol precipitated and washed. The dried pellet was re-suspended in TE buffer (10 mM Tris HCl pH 7.5, 2 mM EDTA) and the DNA concentration was measured using a Qubit 2.0 Fluorometer with the dsDNA BR Assay kit (Invitrogen).

### Reduced Representation Bisulfite Sequencing

Since we previously showed that HM bulls reached puberty one month earlier than MM bulls (Byrne et al., 2018a), the effect of diet and/or earlier puberty was assessed by comparing groups with either identical chronological age (HM15 vs. MM15 comparison) or similar physiological age (HM15 vs. MM16). Reduced Representation Bisulfite Sequencing (RRBS) libraries from HM15, MM15, and MM16 samples were prepared from 200 ng of genomic DNA digested with MspI (Fermentas), as described elsewhere (Gu et al., 2011; Perrier et al., 2018), with the exception that magnetic bead-based size selection was performed using SPRIselect magnetic beads (Beckman-Coulter). Briefly, after ligation to 55 bp methylated Illumina adapters for paired-end sequencing, H_2_O was added up to 50 μl, which was followed by the addition of 35 μl magnetic beads. The larger fragments bound to the beads were removed using a magnetic rack according to the manufacturer’s instructions, and 85 μl of supernatant containing the smaller fragments were transferred into a new tube. The addition of 25 μl of fresh beads next allowed the selection of fragments ranging from 150 to 400 bp (40–290 bp genomic DNA fragments + adapters). After washing with 85% ethanol, the DNA bound to the beads was eluted in 20 μl EB buffer (Qiagen). The DNA was then converted twice with sodium bisulfite using the EpiTect bisulfite kit (Qiagen) following the manufacturer’s instructions. Converted DNA was amplified with Pfu Turbo Cx hotstart DNA polymerase (Agilent) using 14 PCR cycles. The libraries were finally purified using AMPure XP beads (Beckman-Coulter) and sequenced on an Illumina HiSeq4000 sequencer to produce 75 bp paired-end reads (Integragen SA). All pipetting steps before the final amplification were carried out using an NGS STARlet liquid handling system with four channels (Hamilton).

### Bioinformatics and Statistical Analyses

**FIGURE 1:**
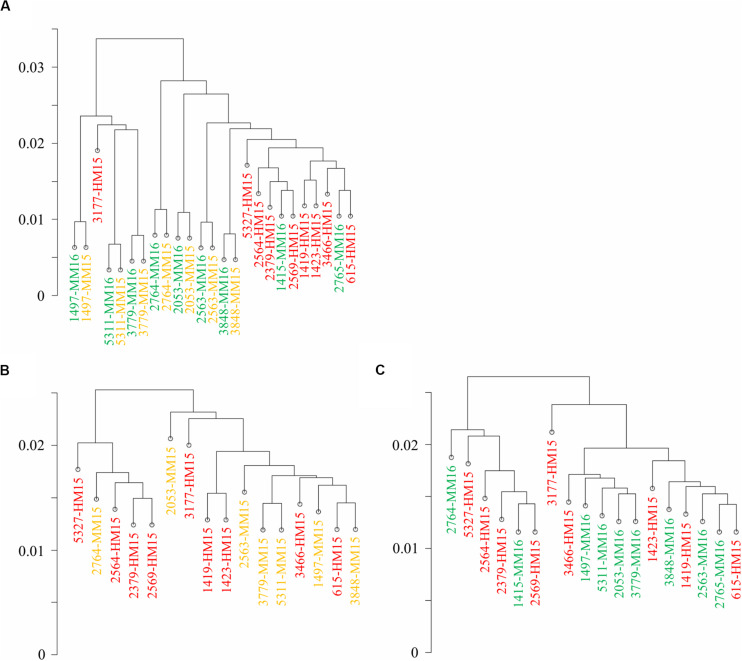
Diet and age do not induce genome-wide modifications of the sperm methylome. Correlation clustering was run on the methylation percentages calculated at CpGs covered more than 10 reads in at least four samples per group. Samples belonging to the three groups are shown in different colors (HM15, red; MM15, yellow; and MM16, green). **(A)** Correlation clustering ran on the whole dataset. Unpaired samples, mainly from the HM group, cluster apart (right part). **(B,C)** Correlation clustering run on unpaired samples from HM15 and MM15 groups **(B)** and HM15 and MM16 groups **(C)**. Taken together, the results demonstrate that the inter-bull variability vs. intra-bull similarity appear to have a greater effect on global bull sperm methylome than any latent effect of dietary regimen *per se*.

The sequences displayed the expected nucleotide composition based on MspI digestion and bisulfite conversion according to FastQC quality control^1^. Subsequent quality checks and trimming were carried out using Trim Galore v0.4.0^2^ which removed adapter sequences, poor quality bases and reads (Phred score below 20) and reads shorter than 20 nucleotides. High quality reads were aligned to the bovine reference genome (UMD 3.1) in which the sequence of the Y chromosome has been incorporated (GenBank: CM001061.2) using Bismark v0.14.3 in the default mode with Bowtie 1 (Langmead et al., 2009; Krueger and Andrews, 2011). The bisulfite conversion rate was estimated from the unmethylated cytosine added *in vitro* during the end-repair step and was ≥97.9%. The CpGs were then selected based on their coverage by uniquely mapped reads. Only CpGs covered by at least 10 uniquely mapped reads (termed as CpGs10) were retained for subsequent analyses. Each CpG10 was assigned a methylation percentage per sample calculated from Bismark methylation calling: [(reads with “C”)/(reads with “C” + reads with “T”)] × 100. HM15, MM15, and MM16 groups were compared for the mapping efficiency, coverage, and average methylation at CpGs10 using permutation tests for k independent samples, with Monte-Carlo sampling of 100,000 permutations. For Figure 1, correlation hierarchical clustering was computed on the matrix of methylation percentages for each CpG10 covered in at least 4 bulls per group. For each comparison, differentially methylated CpGs (DMCs) were identified using DSS v2.14.0 software in the default mode (Feng et al., 2014), and Independent Hypothesis Weighting for p-value adjustment using alpha parameter set to 5% and the average methylation per group as a covariable (Ignatiadis et al., 2016). A CpG10 was considered as a DMC when it was covered in at least 4 bulls per group, when the adjusted p-value was weaker than 0.1, and the methylation difference between two groups at least 10%. A differentially methylated region (DMR) was constituted by a minimum of three DMCs with a maximum inter-DMC distance of 100 bp.

The annotation of the DMCs, DMRs, and the 1,653,793 CpGs10 was performed as described (Perrier et al., 2018) relative to gene features, CpG density and repetitive elements using an in-house pipeline^3^. The reference files were downloaded at the following sites: ftp://ftp.ensembl.org/pub/release-94/gtf/bos_tau rus/Bos_taurus.UMD3.1.94.gtf.gz, http://oct2018.archive.ense mbl.org/biomart/martview/2c4d22063af3b241ef9322512fae0cdc [Ensembl Genes 94, Cow genes (UMD3.1)], http://hgdownload.cse.ucsc.edu/goldenPath/bosTau6/database/cpgIslandExt.txt.gz and http://hgdownload.cse.ucsc.edu/goldenPath/bosTau6/database/rmsk.txt.gz. The following criteria were applied: TSS, -100 to +100 bp relative to the transcription start site (TSS); promoter, -2000 to -100 relative to the TSS; TTS: -100 to +100 bp relative to the transcription termination site (TTS); shore, up to 2000 bp from a CpG island (CGI); and shelf, up to 2000 bp from a shore. A site/fragment was considered to belong to a CGI (respective shore and shelf) if an overlap of at least 75% was observed between the site/fragment and the CGI (respective shore and shelf). A site/fragment was considered as being overlapped by a repetitive element whatever the extent of this overlapping. The list of annotated DMCs and DMRs in the three comparisons are available in Supplementary Table 6. Genes containing DMCs at a maximal distance of 10 kb were subjected to enrichment analyses with the Database for Annotation, Visualization, and Integrated Discovery (DAVID) using default parameters (Huang et al., 2009), and using the gene list containing the 1,653,793 CpGs10 covered in at least four samples per group as the background. Clusters of terms showing EASE enrichment scores above 1.3 were considered significant.

### Validation by Bisulfite-Pyrosequencing

Bisulfite conversion was performed on 0.5 μg genomic DNA using the EpiTect bisulfite kit (Qiagen). Primers were designed using the Pyromark assay design software and amplifications were performed using the Pyromark PCR kit (Qiagen) according to the manufacturer’s instructions. The primers used to amplify each region are listed in Supplementary Table 7. The following PCR program was used: 3 min at 94°C followed by 45 cycles of 30 s at 94°C, 1 min at 56°C, 1 min at 72°C, and finally 10 min at 72°C. The reverse primers were 5′-biotinylated for the DMRs included in *ATP10A*, *NEUROG2*, and *SRGAP3*, and the forward primer for the DMR included in *BCL2*. After denaturation and purification, the biotinylated strand of PCR product was used as a template for pyrosequencing with 0.3 μM pyrosequencing primer, using the Pyromark Q24 device and Pyromark Gold Q96 reagents (Qiagen). The pyrosequencing primers are listed in Supplementary Table 8. Each CpG was analyzed in duplicate, and inconsistent duplicates (more than 5% difference) were repeated. For each sample, the DNA methylation percentage per CpG was obtained by calculating the mean of all replicates that passed quality control by the Pyromark Q24 software. For Figure 4A, correlation significance between the average DNA methylation values per DMR obtained by RRBS and pyrosequencing was tested using Spearman’s rank correlation test. For Figure 4B, HM and MM groups were compared at each CpG using non-parametric tests suited to small samples (permutation tests for two independent samples, with Monte-Carlo sampling of 100,000 permutations and with correction for the stratification of the population according to the age). All statistics were calculated using R software (R Core Team, 2018).

## Results

### Sequencing Quality Controls

In order to examine the effects of plane of nutrition in the first 6 months of life associated with advanced puberty on bovine sperm DNA methylation patterns post-puberty, we analyzed 25 sperm samples from the HM15, MM15, and MM16 groups using RRBS (Supplementary Table 5). Sequencing of the RRBS libraries generated an average of 37 (±6) million reads per sample (Table 1). The average quality score was 38.2 (±0.20), which represents more than 92.4% of bases with a high quality score (Q30 score). The percentage of unique mapping efficiency, although low (on average, 35.3 (±2.7)% uniquely mapped reads), is consistent with what has been previously described in bovine and other ruminant species (Doherty and Couldrey, 2014; Doherty et al., 2016; Perrier et al., 2018), and is due to the significant number of repetitive elements in the bovine genome. Bisulfite conversion rate, monitored using the conversion rate of the unmethylated cytosine added *in vitro* during the end-repair step of library preparation, was on average at 99.4 (±0.7)%. There was no statistically significant difference for any of the above parameters when comparing the different groups of bulls. This eliminated the possibility of technical bias during RRBS library preparation or sequencing that could have affected subsequent results and indicated that the data were of good quality. For the rest of the analysis, we focused on the 58.1 (±4.4)% CpGs that were covered by at least 10 uniquely mapped reads, which we named CpGs10. There was no statistically significant differences between the HM15, MM15, and MM16 groups for the percentages of hypermethylated (DNA methylation >80%), intermediate (DNA methylation in [20–80%]) and hypomethylated (DNA methylation <20%) CpGs10, indicating that neither diet or age difference between 15 and 16 months induce global DNA methylation changes.

**TABLE 1:** Library characterization, mapping efficiency on the bovine genome (UMD3.1), coverage and average methylation in RRBS libraries.

	HM15 (*n* = 9)	MM15 (*n* = 7)	MM16 (*n* = 9)
Number of read pairs (million)	35.1 ± 6.1	36.5 ± 7.0	39.4 ± 7.5
Average quality score (Phred score)	38.3 ± 0.2	38.2 ± 0.3	38.2 ± 0.1
Uniquely mapped reads (%)	35.5 ± 3.1	35.3 ± 2.4	35.0 ± 2.9
Ambiguous reads (%)	52.6 ± 2.8	52.5 ± 3.0	52.7 ± 2.8
Unmapped reads (%)	11.9 ± 1.0	12.2 ± 0.9	12.2 ± 0.8
Bisulfite conversion rate (%)	99.3 ± 0.7	99.4 ± 0.7	99.5 ± 0.6
Number of covered CpGs (million)	3.05 ± 0.1	3.08 ± 0.2	3.08 ± 0.1
Average coverage per CpG	24.8 ± 4.6	25.1 ± 4.4	26.8 ± 4.0
Percentage of CpGs10	56.7 ± 5.0	57.8 ± 4.7	59.8 ± 3.3
Average DNA methylation at CpGs10 (%)	50.3 ± 1.0	50.1 ± 0.9	50.5 ± 0.6
Percentage of CpGs10 hypermethylated (DNA methylation > 80%)	47.1 ± 1.4	46.9 ± 0.7	47.5 ± 0.6
Percentage of intermediate CpGs10 (DNA methylation in [20%; 80%])	7.1 ± 0.5	7.3 ± 0.3	7.1 ± 0.4
Percentage of CpGs10 hypomethylated (DNA methylation < 20%)	45.8 ± 1.1	45.8 ± 0.8	45.5 ± 0.5

*Values are mean ± standard error of the means. CpGs10: CpGs covered by at least 10 uniquely mapped reads. Hypermethylated, intermediate and hypomethylated CpGs10 indicate CpGs10 with average methylation percentage >80%, [20%; 80%], and <20%, respectively.*

#### Inter-Bull Variability Unrelated to Age or Diet Shapes DNA Methylation Profiles

Hierarchical clustering revealed two distinct clusters: one essentially composed of paired samples collected at 15 and 16 months from 3 bulls and another one comprising the other samples including almost all HM15 samples (Figure 1A). However, the clustering of HM samples was not due to the diet. Indeed, six of nine samples from the MM groups clustered together with the HM samples, and the paired MM15/MM16 samples from the same bull all grouped together. Moreover, correlation between independent HM15 samples was lower than that between MM15/MM16 pairs, as reflected by the greater length of the branches separating them on the correlation clustering. To examine the effect of diet and/or advanced puberty more closely, we carried out a new analysis focused on unpaired samples from HM15 and MM15 groups (identical chronological age, Figure 1B), and HM15 and MM16 groups (closer physiological age; Figure 1C). By suppressing the intra-individual effect, the clustering according to the HM diet disappeared. In conclusion, the hierarchical clustering conducted on all CpGs10 did not allow discrimination of samples according to pre-pubertal nutrition or age, but rather emphasized the similarity of semen samples collected at two close stages from the same individual.

#### Stability of Sperm Methylome Between 15 Months and 16 Months of Age

Because the above analyses suggested an important inter-individual variability that could bias the detection of DMCs due to the extreme behavior of some individuals, we next conducted differential analyses using an algorithm described to deal with intra-group inter-individual variability (Feng et al., 2014). To disentangle any confounding effects of age and diet/advanced puberty in the comparison involving HM15 and MM16 samples, we first investigated the impact of the one-month age difference on paired samples of the MM15 and MM16 groups. This resulted in the identification of one unique DMC located in an intron of a gene, *LEMD2* (Supplementary Table 6). This demonstrated that DNA methylation profiles are conserved in semen samples collected at 15 and 16 months of age, a result consistent with the absence of difference reported for semen from sexually mature bulls collected at 12 and 16 months of age (Lambert et al., 2018).

#### Modest Effect of Diet Associated With Advanced Puberty on Sperm Methylome

**FIGURE 2:**
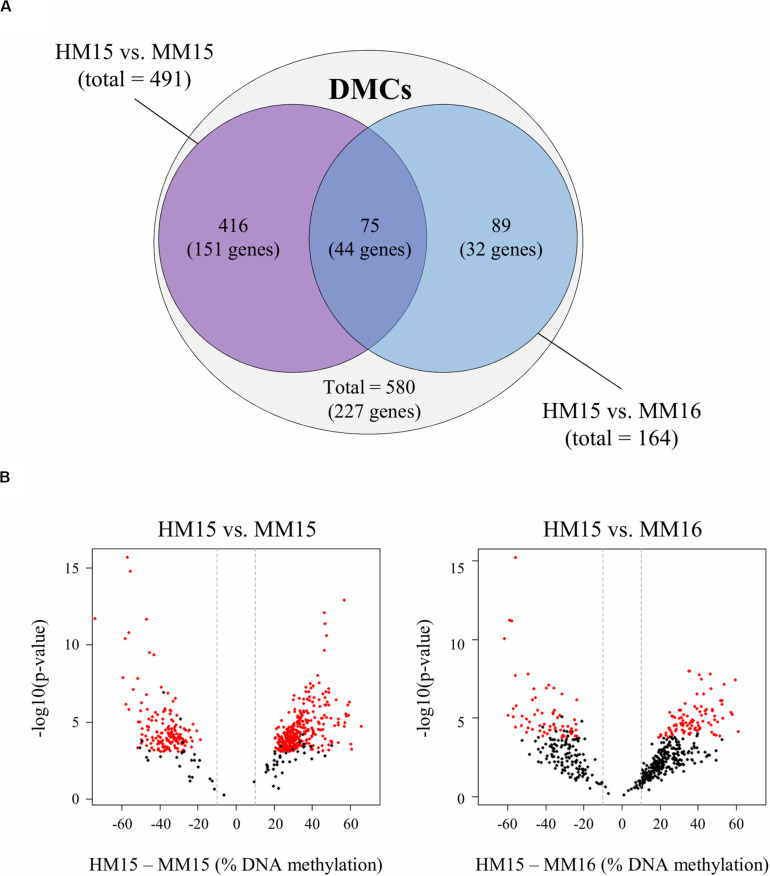
Diet-related differentially methylated CpGs (DMCs) identified in sperm from pairwise comparisons between diet groups. **(A)** Venn diagram showing the 491 and 164 DMCs identified between HM15 vs. MM15 and HM15 vs. MM16 groups, respectively. A total of 580 unique DMCs were obtained. Among those DMCs, 75 (12.9%) are in the middle intersection, representing those that are common between both comparisons. DMCs were associated with 227 unique genes, 44 being common between both comparisons. **(B)** Volcano plots showing the variations of raw *p*-values (*y*-axis, -log10 scale) according to the differences in DNA methylation (*x*-axis, HM – MM differences) for the 580 unique DMCs, in the HM15 vs. MM15 (left) and the HM15 vs. MM16 (right) comparisons. The DMCs that passed the significance thresholds (adjusted *p*-value < 0.1 and difference in methylation ≥ 10%) are indicated in red for each comparison. Most of the 580 unique DMCs, including those that did not reach significance in the HM15 vs. MM16 comparison, showed a similar behavior in both comparison, with a difference in DNA methylation above 10% (indicated by the vertical dashed lines) and a distribution shifted towards positive values indicating a higher methylation in the HM group.

We next focused on the latent effect of nutritional regimen employed during calfhood associated with advanced puberty on DNA methylation profiles. Diet-related DMCs and DMRs were identified by pairwise comparisons between HM15 vs. MM15 (identical chronological age), and between HM15 vs. MM16 (similar physiological age; Figure 2A). In the HM15 vs. MM15 comparison, there were 491 DMCs and 20 DMRs identified, from which 67.4 and 95.0%, respectively, were found hypermethylated for the HM15 group. Similar results were obtained with the HM15 vs. MM16 comparison. A total of 164 DMCs and 4 DMRs were identified, with 61.6% of the DMCs, and all of the DMRs, being hypermethylated in the HM15 group. Therefore, even though the number of DMCs and DMRs between the HM15 group and the two MM groups was within the same order of magnitude, we identified more than twice as many DMCs in HM15 vs. MM15 compared to HM15 vs. MM16. Nevertheless, these DMCs exhibit the same behavior, i.e., most being hypermethylated in HM15 (particularly the DMRs).

In order to understand why the overlap between HM15 vs. MM15 and HM15 vs. MM16 comparisons was limited in spite of the absence of DNA methylation differences between 15 and 16 months, we generated volcano plots for 580 unique DMCs representing the totality of the DMCs identified between the two comparisons. Although the raw *p*-values were higher and the differences in DNA methylation lower for the HM15 vs. MM16 comparison, the 580 unique DMCs exhibited a similar behavior in both comparisons, with differences above 10% for most of them and a distribution slightly shifted towards positive values indicating more DNA methylation in the HM15 group (Figure 2B). We therefore concluded that differences in DNA methylation probably exist between the HM15 and MM16 but do not reach significance thresholds.

**FIGURE 3:**
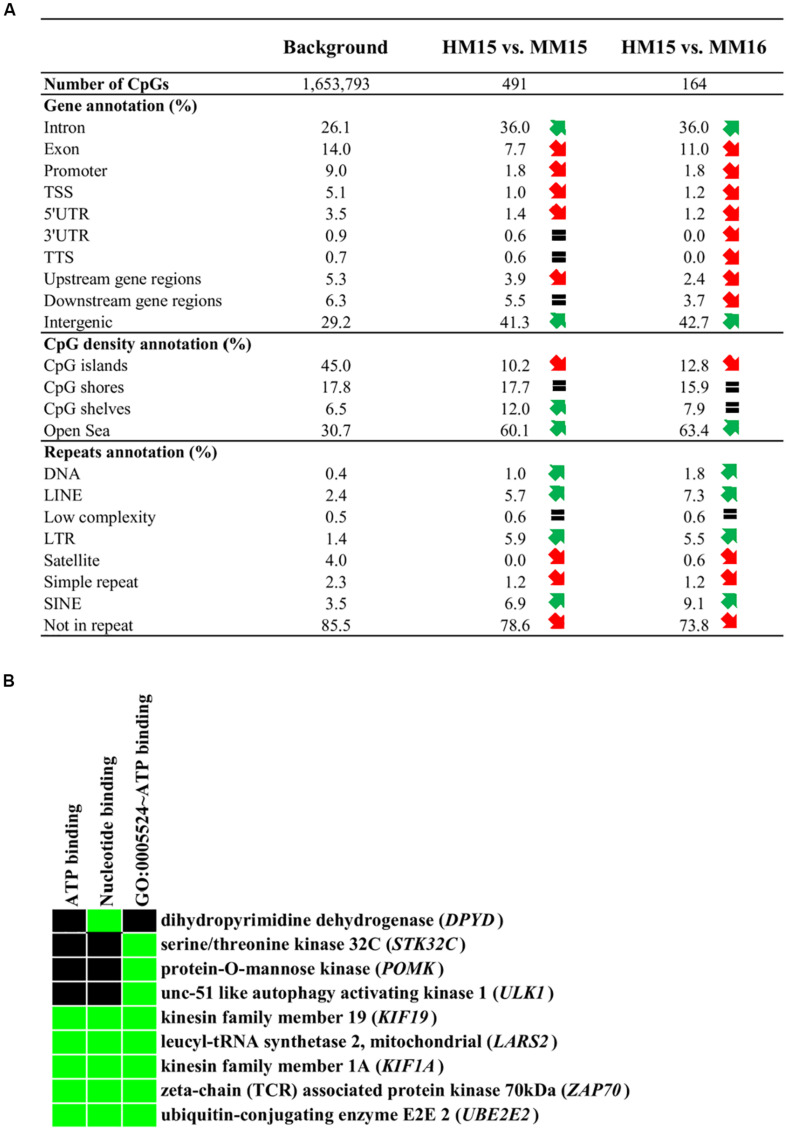
Annotation of the diet-related differentially methylated CpGs (DMCs). **(A)** Annotation of DMCs relative to gene features, CpG density and repetitive elements of the genome. Upstream gene regions (resp. downstream gene regions) represent DMCs located 10 kb upstream of the transcription start site (resp. 10 kb downstream of the transcription termination site). Red and green arrows represent a more than 25% decrease or increase in the relative abundance of genomic features compared to the background, respectively. An equal sign represents that the abundance of the element is less than 25% different to the background. **(B)** Results of enrichment analysis using the DAVID functional annotation tool. The analysis has been performed using the genes identified in the intersection between HM15 vs. MM15 and HM15 vs. MM16 comparisons (Figure 2A, 44 genes). The list of all genes covered by RRBS was used as the background (18,020 genes). The cluster corresponding to ATP binding molecular function showed an EASE enrichment score of 1.36, considering that scores above 1.3 are significant (Huang et al., 2009). Terms of gene ontology or Uniprot keywords enriched among the DMCs are indicated, as are the genes present in each category. The green color on the heatmap represents a correspondence between a gene and a term.

To characterize whether specific genomic features are enriched in DMCs, we carried out the annotation of DMCs found for each of the comparisons (Figure 3A). Essentially, DMCs from HM15 vs. MM15 and HM15 vs. MM16 were distributed similarly. There were more DMCs in intronic and intergenic regions, along with a depletion in exons, promoter-TSS and UTRs of genes, compared to the background. There was also a depletion in CGIs, together with an enrichment in areas less dense in CpGs such as CpG shores, shelves (for HM15 vs. MM15), and open sea. These results are consistent with the fact that only 17.5% of HM15 vs. MM15 and 9.2% of the HM15 vs. MM16 DMCs participate in the formation of DMRs, meaning that the majority of DMCs are dispersed over the genome. Overall, DMCs tended to be more enriched in repetitive elements compared to the background (78.6 and 73.8% of DMCs not associated with repeats for HM15 vs. MM15 and HM15 vs. MM16, respectively, compared to 85.5% for the background), such as LINEs, SINEs and LTRs. On the contrary, DMCs were found depleted in satellites and simple repeats. The lists of DMCs/DMRs, together with their annotation regarding gene features, repetitive elements and CGIs, are available in Supplementary Table 6.

**FIGURE 4:**
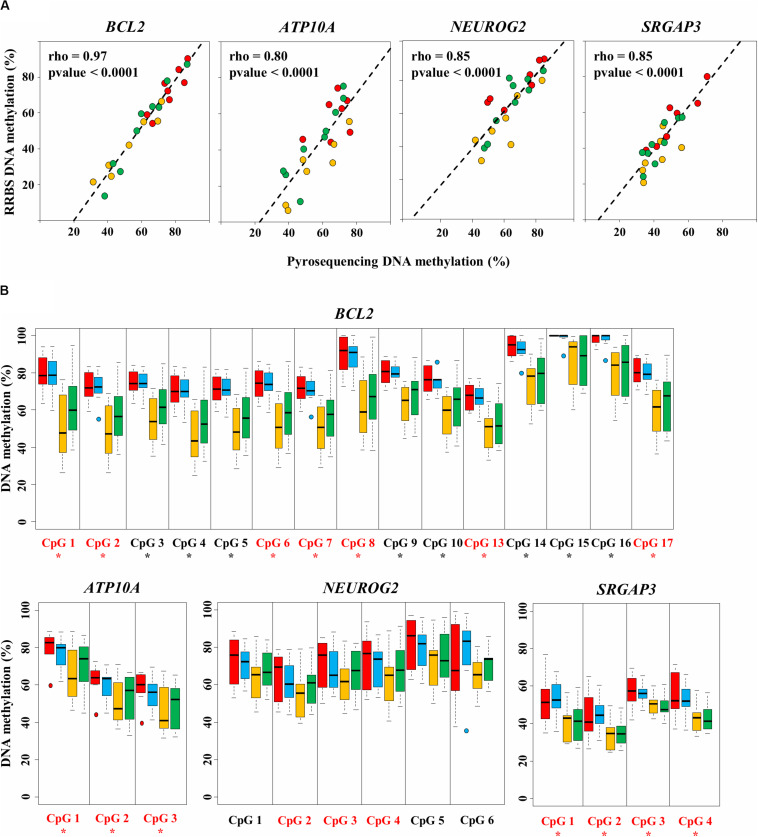
Validation of four diet-related differentially methylated regions (DMRs) by bisulfite-pyrosequencing. **(A)** The average methylation rate measured by RRBS is calculated for the differentially methylated CpGs (DMCs) included in the DMR and plotted against the average methylation rate measured by pyrosequencing. Each dot represents one sample from the HM15 (*n* = 7, in red), MM15 (*n* = 7, in yellow), and MM16 (*n* = 9, in green) groups. The least squares lines of best fit and Spearman’s rank correlation coefficients rho are indicated. All correlations were highly significant (Spearman’s rank correlation test; *p* < 0.0001). **(B)** Methylation percentages of individual CpGs assayed by pyrosequencing in HM15 (*n* = 7, in red), HM16 (*n* = 7, in blue), MM15 (*n* = 7, in yellow), and MM16 (*n* = 9, in green) groups. For each box, the middle line indicates the median and the edges the 25th/75th percentiles. CpGs are numbered according to their 5′–3′ position along the genome. CpGs found as DMCs by the RRBS analysis are shown in red. Asterisks indicate CpGs at which the methylation percentage measured by pyrosequencing is significantly different between HM and MM groups (*p* < 0.05, permutation test corrected for the stratification according to the age).

We then investigated whether specific Gene Ontology (GO) terms were enriched in genes displaying a different representation in DMCs and background. To this end, genes associated with DMCs were subjected to DAVID analysis. While HM15 vs. MM16 exhibited no specific enrichment, for HM15 vs. MM15 an enrichment just below statistical significance was found for a cluster of terms related to ATP binding (EASE score of 1.2; data not shown). Interestingly, using the intersection between HM15 vs. MM15 and HM15 vs. MM16 comparisons (Figure 2A), which accounted for only 44 unique genes, this enrichment became significant (EASE score of 1.36; Figure 3B).

We also performed an extensive review of the literature for all genes differentially methylated between the HM15 and MM15 samples with Biomart annotation available (“Gene name” column in Supplementary Table 6; 174 of 195 genes). Genes relevant to spermatogenesis, sperm function, sexual precocity, gonadal development, and Sertoli cell function and proliferation represented a significant part of all differentially methylated genes (61 over 174) and are listed, together with the corresponding references, in Supplementary Table 9. Strikingly, among these 61 genes, only 6 still retained a methylation difference above significance thresholds in the HM15 vs. MM16 comparison. This is significantly lower than expected by chance (*p* = 0.02; Chi-squared test). Together with the DAVID analysis, these results suggest that the genes differentially methylated in the two HM vs. MM comparisons are not involved in the same biological functions. Genes differentially methylated when HM and MM bulls were compared at 15 months of age are involved in functions that may reflect the different timing of puberty onset in the two diet groups. Considering the genes still differentially methylated when the MM bulls reached a similar level of sexual maturity, these puberty-related functions became less represented and an enrichment in GO terms related to ATP binding could be observed.

#### RRBS Data Validation by Pyrosequencing

To maximize the number of CpG sites to validate by pyrosequencing, we chose to focus on DMRs (as they each contain multiple DMCs). Among the 20 DMRs identified between HM15 and MM15 groups, eight were associated with six genes (the gene *BCL2* exhibiting three DMRs). Four of these six genes are characterized on the bovine genome and are already described in the literature, and therefore were selected for validation by pyrosequencing: ATPase Phospholipid Transporting 10A (*ATP10A*, related to ATP binding GO term), B-cell CLL/lymphoma 2 (*BCL2*), Neurogenin 2 (*NEUROG2*), and SLIT-ROBO Rho GTPase activating protein 3 (*SRGAP3*). For each sample belonging to the HM15, MM15, and MM16 groups, correlations were calculated between the mean DNA methylation of the DMCs composing DMRs on RRBS, to its pyrosequencing counterparts (Figure 4A). We found a significant correlation between RRBS and pyrosequencing data in all of the analyzed regions (0.80 ≤ rho ≤ 0.97; *p* < 0.0001; Spearman’s rank correlation test). This demonstrated that there was a very good agreement between RRBS and pyrosequencing data and validated the data from a technical perspective.

The DNA methylation status of individual DMCs and CpGs included in the DMRs was measured by pyrosequencing (not only on samples from the HM15, MM15 and MM16 groups that were analyzed by RRBS, but also on samples from the HM16 group; Supplementary Table 5). Significant DNA methylation differences between HM and MM groups were identified for genes *ATP10A*, *BCL2*, and *SRGAP3*, for all of the assessed DMCs, validating the RRBS data (*p* < 0.05, permutation test; Figure 4B), while the differences did not reach significance for *NEUROG2*. For all four genes, pyrosequencing analyses confirmed the lower DNA methylation level in the MM groups at each DMR analyzed and the absence of significant change between 15 and 16 months. For the DMRs included in *BCL2* and *ATP10A*, the HM15 and HM16 groups showed a rather homogenous behavior, with similar median DNA methylation percentages and dispersions (indicated by the size of the boxplot). In contrast, the median DNA methylation percentage was slightly increased in the MM16 group compared with the MM15 group, both MM groups exhibiting more dispersion than the HM groups. This could indicate an evolution of the DNA methylation profile at these loci between 15 and 16 months, in some, but not all, individuals of the MM groups. Consistent with the detection by RRBS of only one DMC in the MM15 vs. MM16 comparison, this tendency towards an increased DNA methylation in MM16 group did not reach statistical significance.

## Discussion

This study demonstrates how early life plane of nutrition associated with advancement in the age of puberty can induce changes in the post-pubertal sperm DNA methylation profile.

A rise in luteinizing hormone (LH), typically occurring between 10 and 20 weeks of age, is an important factor in determining the age at onset of puberty in bulls (Rawlings and Evans, 1995). This initial rise in LH secretion is necessary for differentiation and maturation of testicular Leydig cells, leading to the secretion of testosterone. Secretion of follicle stimulating hormone (FSH) is high post-natally and plays a role in Sertoli cell differentiation, reaching a nadir at 25 weeks of age (Brito, 2014). These mechanisms are essential for the onset of spermatogenesis with sperm usually first observed at 32 weeks of age (Amann, 1983). Therefore, augmentations to nutrition during the first months of life have the potential to affect the future functionality of cells that differentiate during this period. In particular, the total number of Sertoli cells is essentially determined by these events, which has direct consequences on the efficiency of spermatogenesis given that each Sertoli cell can support a definite number of germ cells. In a previous study using the same animal model, it was shown that an enhanced plane of nutrition during the first 6 months of life hastened onset of puberty and sexual maturation by approximately one month in the HM group (Byrne et al., 2018a). The hastened onset of puberty was accompanied by an increase in scrotal circumference and in testosterone concentration (Byrne et al., 2018b), indicating that HM bull testes matured earlier. In addition, at 4 months of age, calves subjected to a high plane of nutrition had greater seminiferous tubule diameter, more mature spermatogenic cells and more Sertoli cells, compared to calves on a low plane of nutrition (English et al., 2018). In agreement with the increased testosterone concentration observed at a later age, calves in the high plane group also exhibited a tendency for greater peripheral LH concentrations. They also exhibited 1,346 differentially expressed genes in the testicular parenchyma, most of which were upregulated and involved in the production of androgen or cholesterol biosynthesis. However, once all animals were sexually mature no subsequent modifications in either semen volume or quality were evident when compared to the MM group (Byrne et al., 2018a). These phenomena demonstrate that the enhanced plane of nutrition hastened sexual maturation and the onset of puberty without resulting in obvious latent effects on traditional measures of sperm quality. In contrast, in the present study we observed that HM and MM bulls can be distinguished based on their sperm DNA methylation profile after puberty.

The first observation is that more DMCs, a higher proportion of which were hypermethylated in HM bulls, were identified between the HM15 vs. MM15 groups than between the HM15 vs. MM16 groups, which could be related to the advanced maturity of HM bulls rather than to the diet early in life. Indeed, the MM15 and MM16 samples originated from animals on the same diet; therefore, if the DNA methylation changes solely reflected the direct effect of diet, a similar number of DMCs, with a similar proportion of hypermethylated DMCs would have been expected in the two comparisons between HM and MM bulls. Furthermore, the differential methylation observed between HM15 and MM15 but not between HM15 and MM16 samples targets genes relevant to puberty and may be related to the more advanced maturity of the HM bulls, which is counterbalanced when MM bulls are used one month later. In contrast, the differential methylation still observable in the HM15 vs. MM16 comparison targets other genes and may reflect a long-term memory of the metabolic and physiological consequences induced by HM diet and advanced puberty, that may persist at later stages. The hypothesis that advanced puberty is the main determinant of the DNA methylation patterns we report herein is further supported by the gain of DNA methylation observed in the sperm of sexually mature bulls at 16 months of age compared to when they were 10 months old (Lambert et al., 2018). The quasi absence of DMCs when MM animals were compared at 15 and again at 16 months of age is also consistent with the relative stability of the sperm methylome in sexually mature bulls reported by Lambert et al. (2018). Importantly, it indicates that the lower number of DMCs and proportion of hypermethylated DMCs in the HM15 vs. MM16 comparison (relative to the HM15 vs. MM15 comparison) can be attributed to the difference in sexual maturity rather than in age. Indeed, the MM bulls were already sexually mature at 15 months of age and interval to the second time point (1 month later) may have been too short in duration to expose significant age-related differences, should they exist. Why then, if MM15 and MM16 samples are similar in terms of DNA methylation pattern, did the two comparisons involving HM and MM bulls give rise to different amounts of DMCs, with a limited extent of overlap between the two sets of DMCs? A possible explanation may be related to the experimental design. Only paired samples were considered for the comparison between 15 and 16 months of age, therefore excluding any influence of genetic factors on the compared DNA methylation profiles. In contrast, for the HM vs. MM comparisons, independent samples were used, with no complete overlap between the MM15 and MM16 groups in terms of included bulls due the unavailability of the biological material. Given the limited number of individuals included in this study, we can therefore not exclude that DNA methylation differences at some DMCs rely on genetic polymorphisms, as has been described in humans (Lappalainen and Greally, 2017), and in agreement with the significant inter-bull variability highlighted by the hierarchical clustering run on our RRBS data.

Although the algorithm used to detect differential DNA methylation is highly stringent compared with others (Feng et al., 2014), the number of DMCs identified between HM and MM groups could be considered as modest, with a total of 580 DMCs relative to the 1,653,793 CpGs covered by the RRBS method. However, the genes targeted by differential methylation have compelling relationships with testis development and puberty. Based on these genes, four potential explanations for the advanced sexual maturity of HM bulls induced by enhanced nutrition are detailed below. Firstly, four differentially methylated genes are found in regions with genome-wide association study (GWAS) signals associated with sexual precocity in Nellore cattle (Irano et al., 2016). *ATP10A*, related to the ATP binding GO term that was enriched in our study, is located in a region associated with early pregnancy. Even more interestingly, *SEC23B*, *SRGAP3*, and *LHFPL4* are located in regions associated with scrotal circumference, a trait specifically modified in HM bulls. Of note, *ATP10A* and *SRGAP3* account for two of the 20 DMRs we identified between HM15 and MM15 samples and were analyzed more precisely and validated by pyrosequencing. *ATP10A* is known to be imprinted in humans, but this specificity has not been described in cattle. Interestingly, a lack of *ATP10A* activity in adulthood is related to obesity in humans (Gillessen-Kaesbach et al., 1999) and *SRGAP3* expression is known to be influenced by its DNA methylation status (Gao et al., 2016). Together with our results, these reports indicate that *ATP10A* and *SRGAP3*, as putative candidate genes for sexual precocity, are regulated not only by genetic factors but also by environmental and epigenetic factors. The second compelling piece of evidence for the involvement of the differentially methylated genes in sexual maturity of HM bulls is provided by a study using Yiling goats, a native Chinese breed exhibiting precocious puberty, as a model to investigate the changes in the testis transcriptome that parallel the post-natal development of testis (Bo et al., 2020). In that study, 120 days of age was identified as the transition point at which testis weight dramatically increases and spermatogenesis becomes functional in Yiling goats; two genes targeted by differential methylation in our study, *ALDOB* and *PIGR*, are listed as “hub genes” whose expression is modified during this transition. Although not listed as “hub genes” by Bo et al. (2020), we identified several genes involved in spermatid differentiation or highly expressed post-meiotically, such as *RARB*, *ADCY8*, and *OSBP2* (relevant references are provided in Supplementary Table 9). We also identified many differentially methylated genes involved in cytoskeleton remodeling, cilium growth and cell junction, such as *ACTN1*, *GSN*, *MTMR7*, *PACRG*, *TUBB2B*, and *ARC*. Interestingly, the genes differentially expressed during the transition to functional spermatogenesis in Yiling goats are involved in similar functions, suggesting that the methylation changes between HM and MM bulls are the consequence of a different dynamics in the process of testis maturation around puberty. The third potential explanation for the hastened puberty of HM bulls involves the hypothalamic-pituitary-gonadal axis, which is crucial for puberty and reproductive function. Indeed, our study points to genes directly acting at different levels of this axis, such as *PREP*, *WWOX*, and *CALB2*. *PREP* encodes a prolyl endopeptidase that metabolizes gonadotropin-releasing hormone (GnRH) in the hypothalamus and pituitary gland and could contribute to pulsatile GnRH secretion that precedes the onset of puberty (Yamanaka et al., 1999). The *PREP* gene product is detected in both somatic cells and germ cells of the testis, as well as in sperm; *PREP*-deficient mice exhibit smaller testes, abnormal spermatogenesis and decreased sperm motility (Dotolo et al., 2016). *WWOX*-deficient mice die before puberty, and exhibit reduced expression of *LH* and *FSH* genes in the pituitary gland, abnormal testis development and alteration in the number and size of Leydig cells (Aqeilan et al., 2009). Overexpression of *CALB2* in mouse primary Leydig cells upregulates testosterone production in response to LH signaling (Xu et al., 2018). The differential methylation of these genes may thus reflect a higher degree of stimulation of the hypothalamic–pituitary–gonadal axis in the HM bulls. The fourth potential explanation for hastened testis development in response to enhanced nutrition in HM bulls is related to the balance between proliferation, survival and apoptosis in Sertoli and germ cells, which is highly sensitive to the nutritional status. Indeed, *BCL2*, a well-known antiapoptotic gene that enhances anti-oxidant enzyme activities (Hockenbery et al., 1993), was identified as differentially methylated in our study, as well as two other genes for which a role in the regulation of apoptosis in germ cells has been reported, *CAST* and *NFIX* (references in Supplementary Table 9). Differential methylation affects *BCL2* at three regions, among which one has been analyzed in more detail by pyrosequencing. In Sertoli cells of immature animals, *BCL2* contributes to the control of cell proliferation through estrogen signaling (Lucas et al., 2011). Estrogens are synthesized from androgens by the aromatase encoded by *CYP19* that is stimulated by FSH, therefore bridging the hypothalamic-pituitary-gonadal axis with the control of apoptosis in Sertoli cells. *BCL2* is also highly expressed in Sertoli cells of sexually mature animals, which may be essential to promote survival of these cells since they are essentially quiescent after puberty and cannot be replaced (Aslani et al., 2017). Interestingly, in sexually mature sheep exposed to undernutrition, spermatogenesis reversibly regresses due to the alteration of Sertoli cell function that in turn induces apoptosis in germ cells (Guan and Martin, 2017). Likewise, in the sperm of mice fed a low protein diet, genes involved in apoptosis were found hypomethylated, echoing the hypermethylation we report here in the HM groups (Watkins et al., 2018). These reports highlight the link between metabolic status and apoptosis in Sertoli and germ cells. As well as the control of apoptosis, autophagy may also contribute to the larger number of Sertoli cells at a younger age and to the hastened onset of puberty of HM bulls, as suggested by the differential methylation status we observed in two key genes involved in this pathway, *ULK1* and *TFEB*. Autophagy is a stress response pathway that promotes cell survival by providing energy and nutrients through the self-digestion of cellular constituents by lysosomes. *ULK1* encodes a protein kinase that triggers the entry of the cell into the autophagy pathway upon starvation and therefore bridges together energy metabolism with autophagy. Pre-pubertal energy restriction in sheep induces testis autophagy and apoptosis by respectively increasing *ULK1* gene and protein expression and modifying the ratio between *BCL2* and its pro-apoptotic antagonist *BAX* (Pang et al., 2018). *TFEB* encodes a transcription factor that activates the transcription of many genes involved in lysosome biogenesis and autophagy and its expression is tightly regulated during spermatogenesis. During the differentiation of spermatogonia, the activation of autophagy by TFEB facilitates migration towards the lumen of seminiferous tubules through the recycling of adhesion molecules, receptor and matrix proteins (Liu et al., 2018). In contrast, the induction of the meiosis gatekeeper *STRA8* at the onset of meiosis is associated with a repression of autophagy and with *ULK1* and *TFEB* downregulation (Ferder et al., 2019). In Sertoli cells, *TFEB* mediates the initiation of autophagy upon exposure to toxins such as ethanol, in order to promote cell survival and sustain spermatogenesis (Horibe et al., 2017). Interestingly, *ULK1* directly relates autophagy with the mTOR pathway, which regulates many metabolic processes and essential cell functions in response to the nutrient and energy status of the cell (Kim et al., 2011). *RPTOR*, an important player in the mTOR pathway targeted by differential methylation between HM and MM bulls, is necessary for the maintenance and self-renewing population of spermatogonial stem cells, and *RPTOR*-deficient mice are infertile (Serra et al., 2019).

A question to be addressed is the potential impact of these DNA methylation modifications on the phenotype in subsequent generations. Changes in both DNA methylation patterns and expression of genes involved in energy metabolism have been recently reported in embryos generated using the sperm from peripubertal bulls (10 and 12 months of age), relative to those obtained with sperm collected at 16 months of age from the same bulls (Wu et al., 2020). This raises the possibility that part of the methylation changes in HM bulls, which reached sexual maturity earlier than MM bulls, may influence embryo metabolism, which may also have important consequences at later stages of development. For an alteration of DNA methylation pattern to have an effect on gene expression during embryonic development or on offspring phenotype, the alteration needs to escape or to interfere with the reprogramming process occurring after fertilization. Imprinted genes, but also some endogenous retroviruses, escape reprogramming during early embryo development (Seah and Messerschmidt, 2018). In the mouse, a subset of LINE and LTR elements, as well as intergenic regions, are highly methylated in sperm cells and resist genome-wide DNA methylation erasure in the embryo (Wang et al., 2014). It is interesting to note the DMCs we identified were enriched in these genome features, raising the possibility that the hypermethylation observed in HM sperm samples is transmitted to the embryo. Based on these reports, further analyses on DNA methylation patterns and gene expression on embryos from bulls that had undergone hastened puberty onset, and of the resultant offspring, is warranted.

In conclusion, the data presented here demonstrate that a high plane of nutrition during the first 6 months of life in bull calves associated with an advancement in the onset of puberty induces modest latent modifications of the sperm methylome after puberty. Evidence suggest that these alterations of the sperm methylome reflect advanced puberty onset of the calves rather than plane of nutrition *per se*, with the differences in DNA methylation patterns being reduced when bulls on a high plane of nutrition were compared to older animals on a moderate plane of nutrition. These findings should be replicated on an independent and larger cohort of bulls. The modification of sperm DNA methylation does not appear to have any significant effect on sperm functional parameters routinely assessed pre- and post-thawing in artificial insemination centers; further work needs to be performed to assess any latent effects on fertilizing ability, embryo development, as well as on offspring health.

## Data Availability Statement

The datasets generated for this study can be found in the European Nucleotide Archive (ENA) at EMBL-EBI under accession number PRJEB35837 (https://www.ebi.ac.uk/ena/data/view/PRJEB35837).

## Ethics Statement

All protocols were in accordance with the Cruelty to Animals Act (Ireland 1876, as amended by European Communities Regulations 2002 and 2005) and the European Community Directive 86/609/EC. All animal procedures performed were conducted under experimental license from the Irish Department of Health and Children.

## Author Contributions

J-PP participated in the pyrosequencing experiment, RRBS and pyrosequencing data analysis, and drafted the manuscript. DK, SF, and PL conceived the study and performed critical revision of the manuscript. AC-T performed the RRBS and pyrosequencing experiments. CB participated in the conception of the study, collected the samples, and participated in drafting the manuscript. ES performed experiments and participated in the conception of the study and in the critical reading of the manuscript. LJ and AA-F performed the bioinformatics and statistical analyses of RRBS data. LS and HJ obtained funding and participated in the conception of the study and edited the manuscript. HK coordinated the study and carried out the data analysis and editing of the manuscript. All authors have read and approved the final manuscript.

## Conflict of Interest

The authors declare that the research was conducted in the absence of any commercial or financial relationships that could be construed as a potential conflict of interest.

## Footnotes

1 https://www.bioinformatics.babraham.ac.uk/projects/fastqc/

2 http://www.bioinformatics.babraham.ac.uk/projects/trim_galore/

3 https://github.com/FAANG/faang-methylation/tree/master/RRBS-toolkit/Annotation
